# Benchtop Low-Frequency 60 MHz NMR Analysis of Urine: A Comparative Metabolomics Investigation

**DOI:** 10.3390/metabo10040155

**Published:** 2020-04-16

**Authors:** Justine Leenders, Martin Grootveld, Benita Percival, Miles Gibson, Federico Casanova, Philippe B. Wilson

**Affiliations:** 1 Leicester School of Pharmacy, De Montfort University, The Gateway, Leicester LE8 9BH, UK; justineleenders@gmail.com (J.L.); mgrootveld@dmu.ac.uk (M.G.); benita.c.percival@dmu.ac.uk (B.P.); miles.gibson@dmu.ac.uk (M.G.); 2 Magritek Gmbh, Philipsstraße 8, 52068 Aachen, Germany; federico@magritek.com

**Keywords:** low-frequency NMR, benchtop spectrometers, type 2 diabetes, metabolomics, ^1^H-^1^H COSY

## Abstract

Metabolomics techniques are now applied in numerous fields, with the ability to provide information concerning a large number of metabolites from a single sample in a short timeframe. Although high-frequency (HF) nuclear magnetic resonance (NMR) analysis represents a common method of choice to perform such studies, few investigations employing low-frequency (LF) NMR spectrometers have yet been published. Herein, we apply and contrast LF and HF ^1^H-NMR metabolomics approaches to the study of urine samples collected from type 2 diabetic patients (T2D), and apply a comparative investigation with healthy controls. Additionally, we explore the capabilities of LF ^1^H-^1^H 2D correlation spectroscopy (COSY) experiments regarding the determination of metabolites, their resolution and associated analyses in human urine samples. T2D samples were readily distinguishable from controls, with several metabolites, particularly glucose, being associated with this distinction. Comparable results were obtained with HF and LF spectrometers. Linear correlation analyses were performed to derive relationships between the intensities of 1D and 2D resonances of several metabolites, and R^2^ values obtained were able to confirm these, an observation attesting to the validity of employing 2D LF experiments for future applications in metabolomics studies. Our data suggest that LF spectrometers may prove to be easy-to-use, compact and inexpensive tools to perform routine metabolomics analyses in laboratories and ‘point-of-care’ sites. Furthermore, the quality of 2D spectra obtained from these instruments in half an hour would broaden the horizon of their potential applications.

## 1. Introduction

High-frequency (HF) ^1^H NMR analysis has been successfully developed to be applied in untargeted metabolomics investigations, through decades of successive optimisations with a wide range of biological media [1]. Metabolomics comprises both quantitative and qualitative analyses of whole metabolomes, which arise from a complex series of metabolic interactions and processes occurring within cells, tissues or organs. The overall purpose of metabolomics investigations is to detect, quantify and interpret variations in the concentrations of metabolites responsible for biological behaviour; the detection of perturbations or imbalances in metabolic pathways can reveal defects in the function or activities of selected enzymes therein, which may be characteristic of particular disease processes [2]. The detection of such defects may therefore lead to the identification of potential biomarkers, or the development of targeted diagnostic test systems for the diagnosis and prognosis of diseases, processes which also give rise to improved understandings of their pathologies. This, in turn, may also lead to the identification of suitable drug targets [3].

Moreover, in addition to pathologies, such approaches may also provide a complete and systematic profiling of metabolites and their temporal changes caused by factors such as diet, lifestyle, environment and administered drugs. This is possible through the multicomponent analysis of body fluids and/or extracts of tissue biopsy samples, coupled with a statistical interpretational strategy known as multivariate analysis [4]. In view of its multicomponent analytical advantages, HF NMR analysis serves as a powerful tool for the simultaneous and rapid identification and quantification of large numbers of biomolecules present in biofluids or tissue sample extracts. Hence, it is ideal for probing the metabolic profiles of complex samples collected from living systems, for diagnostic or prognostic purposes [5,6].

HF NMR spectroscopy has long been employed to conduct metabolomics studies, and offers many further significant bioanalytical advantages for such applications. Indeed, the technique is virtually non-destructive, and requires a short analysis time, and for biofluids, usually requires very little sample preparation [7]. However, HF NMR analysis also presents a number of disadvantages, such as cost, size, and transportation limitations, and often the requirement for specialist technical staff for instrumental operation. Moreover, the size of such high-resolution NMR facilities limits their accessibility to many researchers, and therefore precludes regular use outside of university-based NMR-dedicated laboratories, along with those in large industrial centres.

Low-frequency (LF) applications of NMR have previously been explored in some detail [8,9,10], and the future of NMR-based metabolomics has also been recently discussed, with an emphasis on this approach providing a convincing solution for metabolic fingerprinting at ‘point-of-care’ sites or prospectively, even for personal use [11,12,13]. Moreover, LF ^1^H NMR applications have recently been shown to distinguish between control and type 2 diabetic (T2D) participants, with potential use for point-of-care applications in a metabolomics study using a near-portable benchtop NMR spectrometer as a novel bioanalytical tool [11]. The advantages of this technique included fast acquisition time, ease-of-use and low limits of detection [11]. Moreover, LF benchtop NMR facilities do not require expensive cryogens, nor a Gauss safety line for the magnetic field, and much less power is required for operation, achieving desirable low running costs for potential applications in clinical chemistry. However, although acceptable correlations between urinary metabolite calibration standard concentrations, and selected prominent metabolites detectable in urine itself, particularly those of glucose, were found between the LF and HF analyses conducted, the overall metabolomics results obtained in this study at an operating frequency of 60 MHz were not directly validated by comparisons with those arising from the application of a corresponding HF NMR facility. f especial interest is an evaluation of whether the nature of detectable metabolites, and the number of these found as discriminant markers in the 60 MHz study, were comparable to those found using HF NMR analysis. Furthermore, disadvantages of the one-dimensional (1D) approach included a lack of resolution, or more specifically a range of resonance overlap and crowding problems encountered in LF analysis. Indeed, it was not possible to resolve the β-glucose anomeric proton signal from the residual water resonance. However, the use of such 1D LF techniques for many qualitative analyses, as well as the development of appropriate and robust automated computational tools for real-time analysis, have the potential to impact on a myriad of additional applications.

In this pilot study, we first investigate LF and HF datasets robustly, in order to compare the accuracies and precision of statistical tools via the acquisition of urinary spectra at both operating frequencies. We also present results regarding improvements in spectral resolution, which are achievable via the acquisition of 2D NMR urinary profiles through LF ^1^H-^1^H correlation (COSY) spectra. This paper complements the preliminary metabolomics results reported by Percival et al. [11], and describes for the first time the fully assigned 2D spectra acquired at LF, in addition to a robust comparison between metabolomics studies performed at both operating frequencies. Results presented in this manuscript are therefore sub-divided into three sections. The first phase of the study investigates the capabilities and analytical advantages of 2D NMR spectra acquired at low-field, whilst the second phase compares the results of metabolomics analyses performed at both LF and HF NMR operating frequencies. The final phase is based on the results obtained from Phase 2, and, partial least square (PLS)-regression analysis, is conducted in order to determine the relationship between glucose concentrations detectable in the urinary ^1^H NMR profiles of T2D patients, and also modifications in the urinary excretion of other metabolites.

## 2. Results

### 2.1. Analysis of 2D “POWER COSY” 60 MHz LF Spectra

**Figure 1:**
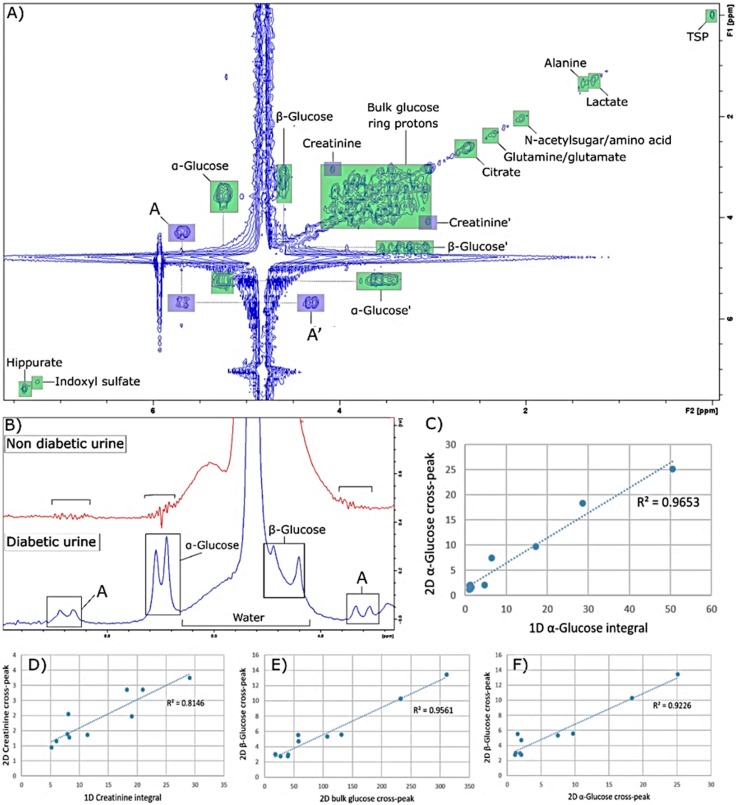
Investigation of 2D ^1^H-^1^H COSY low-frequency NMR spectra. (**A**): 2D ^1^H-^1^H COSY NMR T2D urinary profile acquired at 60 MHz using a benchtop NMR facility. Creatinine blue squares represent the long-range connectivity cross-peak for this metabolite. Blue squares labelled A represent unassigned, unusual doublet resonances arising from ‘mirroring’ spectral signal located at δ = 5.13–5.29 ppm (in this case, reflecting the α-glucose cross-peak). (**B**): Unassigned 4.38 and 5.70 ppm doublet resonances, detectable in the 1D 60 MHz NMR profile of a urine sample collected from a hyperglycaemic T2D patient, but completely absent from that of a healthy control participant. Urea, which is observable in HF 400 MHz spectra and resonates at ~5.80 ppm, was not readily observable in our LF spectra in view of its broadness and overlap with the unassigned δ = 5.70 ppm signal. Typical spectra are shown. (**C**,**D**): Linear correlations between 1D and 2D integrals of α-glucose’s C1-H 5.25 ppm resonance (**C**), and that of creatinine’s N-CH_3_ function at δ = 3.06 ppm (**D**). (**E**,**F**): Linear correlations between 2D cross-peaks signal integrals of β-glucose’s C1-H signal versus those of glucose bulk ring (**E**), and α-glucose’s C1-H function (**F**).

2D NMR measurements were acquired on 10 T2D patient urine samples, using gradient- enhanced magnitude for ^1^H-^1^H COSY experiments. As shown in Figure 1, at least several metabolites can be identified via their corresponding diagonalized resonances, and correlation signals are observed for those present at high urinary concentrations, such as glucose, as well as a four-bond connectivity (long-range connectivity cross-peak) for creatinine at [3.06,4.06] ppm. Interestingly, apparent doublet resonances were observable in the LF one- and two-dimensional NMR spectra, but not in the corresponding HF equivalents. Indeed, two regions of the spectrum, at δ = 5.55–5.72 and 4.24–4.40 ppm, appeared to be “mirrored” by a specific region of the spectrum within the 5.13–5.29 ppm range. In T2D patients, with the doublet at 5.25 ppm assigned to the α-glucose anomeric C1-H proton, these signals appear in 1D spectra as a “mirroring” doublet of the α-glucose anomer’s signals located at δ = 4.3 and 5.7 ppm, and a corresponding cross-peak signal was observed in the 2D COSY spectrum (Figure 1A,B, respectively). Moreover, it should be noted that the urea resonance located at δ = 5.7 ppm, which is readily detectable in the HF spectra, is much less visible in the 60 MHz profiles in view of this interfering signal, which as expected, is certainly not observed when glucose is not ^1^H NMR-detectable in urine samples (Figure 1B).

Integration of the glucose (α-glucose [5.25,3.58]; bulk glucose ring protons [3.20,3.98] ppm) and creatinine ([3.06,4.06] ppm) 2D cross-peaks signals, and those of corresponding 1D proton intensities, was followed by their normalization to the internal TSP reference signal. Notably, β-glucose proton integration values could not be used for quantification purposes, in view of a significant overlap with the residual water signal at 60 MHz operating frequency. Furthermore, a 1D integration of the creatinine signals was performed on the 3.06 ppm >N-CH_3_ resonance only, since that of the -CH_2_ function at 4.06 ppm was subject to overlap from those arising from glucose bulk-chain protons. Relationships between these 2D cross-peak intensities and those of matched 1D proton signals were investigated by linear correlation analysis. As shown in Figure 1, regression lines show strong correlations between the TSP-normalised 1D and corresponding 2D integrals, with R² values of 0.96, 0.95 and 0.81 for α-glucose, the bulk glucose ring protons, and creatinine (Figure 1C, E and D respectively).

As noted above, the 4.60 ppm β-glucose signal cannot be integrated in 1D spectra, in view of a very high level of overlap with the residual water resonance in this region. However, the 2D cross-peak resonance is clearly observable in 2D COSY spectra acquired, as shown in Figure 1. To assess whether the proximity of the water signal also interferes with the β-glucose intensity in 2D spectra, linear correlation analyses of the intensity of the 2D cross-peak of β-glucose versus those of α-glucose or the bulk glucose ring were performed. Excellent correlations were found, with R^2^ values for the relationships of β-glucose with α-glucose and bulk glucose ring protons being 0.92 and 0.96 respectively (Figure 1E,F respectively). The unusual δ = 4.3 and 5.70 ppm resonances were also strongly correlated with the δ = 5.13–5.29 ppm spectral region (R^2^ = 0.96 and 0.97 respectively), and these data provide evidence for its glucose source. Association between these spectral zones was evaluated by computing the 1D integral ratio of α-glucose with these signals’ integral intensity ratios, i.e., 5.25/5.70 or 5.25/4.38 ppm. Based on this, spectral intensities at the 5.70 ppm integral revealed a mean intensity of 22.4 ± 3.6% (mean ± SEM values) of that at 5.25 ppm, whereas the integral value for the δ = 4.38 ppm resonance was 11.6 ± 1.7% of that at 5.25 ppm. Overall, this analysis confirmed that these three separate spectral zones are strongly correlated. However, although correlated with glucose’s α-anomer C1-H resonance, these two unusual coupled doublet signals (*J* = 4.23 Hz) are not simply explicable, and therefore further experiments are required to establish their precise identity.

In principle, the gradient (regression coefficient) of the plot of the intensity of the β-glucose C1-H anomeric proton 2D cross-peak (δ = 4.66 ppm), versus that of the corresponding 5.25 ppm α-glucose one, should reflect their natural abundancies, i.e., 64%:36% = 1.64. However, this gradient was only 0.60, and this much lower value arises from the β-anomer 1D resonance being much closer to the water presaturation frequency (δ = 4.74 ppm) than that of the α-anomer. The marked influence of the presaturation process on the intensity of the 1D α-glucose resonance has already been explored in detail in Ref. [11], at both 60 and 400 MHz operating frequencies.

### 2.2. Metabolomics Investigation of T2D Versus Healthy Urine Samples: Low- and High-Frequency ^1^H NMR Comparisons

**Figure 2:**
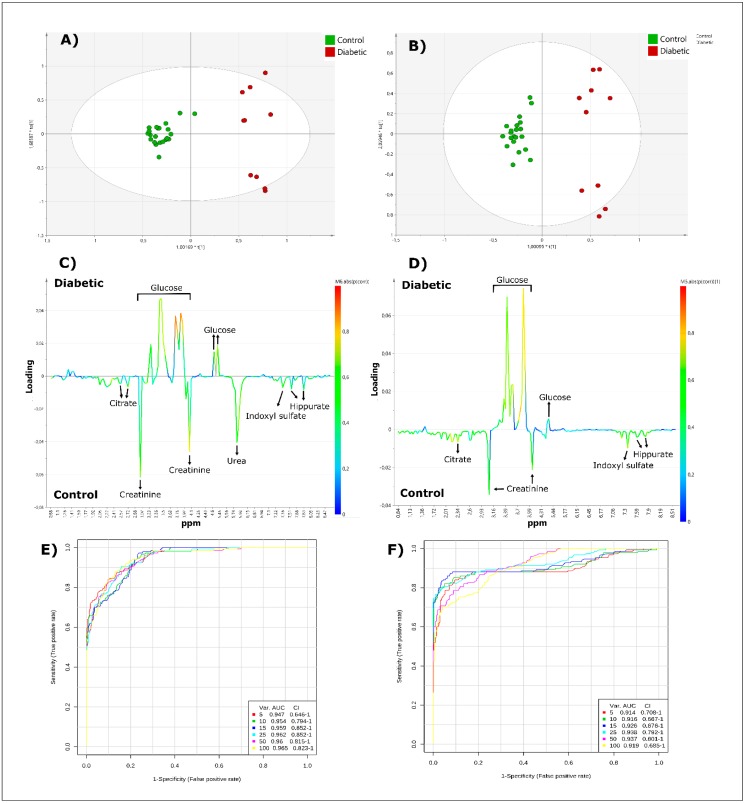
Metabolomics analysis of T2D ^1^H NMR urinary profiles (*n* = 10), versus those of healthy controls (*n* = 15), acquired using HF and LF NMR spectrometers. Scores plots (**A**,**B**), and corresponding loading-plots (**C**,**D**) from orthogonal partial-least squares discriminant analysis (OPLS-DA) applied to ^1^H-NMR spectra of control versus T2D samples, acquired using HF (**A**,**C**) or LF (**B**,**D**) ^1^H NMR analysis. The colour of the signals in the loading plots correspond to the metabolites contributing most greatly towards the separation between T2D and healthy control group samples. The colour bar next to the right-hand side of these plots indicates the level of importance of the metabolites discriminating between classes (either positively or negatively so), the least significant represented by a blue colouration, and the most highly significant metabolites, red. (**E**,**F**): Receiver operating characteristic (ROC) curve (with an area under ROC curve (AUROC) value of 0.96 (**E**, HF NMR acquisition) and 0.94 (**F**, LF NMR acquisition) obtained from the PLS model building system, explored with 25 discriminant variables.

Urinary ^1^H NMR spectra of 10 non-rigorously controlled T2D patients and 15 self-reported healthy control participants were acquired using both 60 and 400 MHz spectrometers. Spectral comparisons between both frequencies, along with a table of identified metabolites, is shown in the Supplementary Material (Figure S1). In order to determine whether the metabolomics analysis performed effectively on spectra acquired on a LF benchtop NMR facility, and provides results comparable to those of a HF instrument, a comparative multivariate statistical analysis of datasets was performed, and the results acquired compared. An orthogonal partial-least squares discriminant analysis (OPLS-DA) scores plot (component 2 versus component 1) featuring both T2D and control participants revealed identical spatial localizations of the patients, under both HF and LF conditions (Figure 2). Samples could be separated into two clear clusters according to their health status along the first PC, and these acquired results appeared to be similar for experiments conducted at both spectrometer operating frequencies (Figure 2A,B). Moreover, analysis of the second component showed a wide *intra*-group variation within diabetic samples, with the appearance of two sub-clusters within this classification. A further analysis revealed that these two sub-clusters arose from diabetic patients presenting with high urinary glucose concentrations, and those with only little or none of this metabolite detectable. The cluster separation of T2D and control samples is clearly observable under both LF and HF conditions, with LF model R² and Q^2^ values of 0.924 and 0.611, respectively, and similar HF model values of 0.943 and 0.706, respectively (for models with a total of three components).

Most relevant variables linked to this separation were extracted from an S-line correlation and VIP plots. In the S-curve, the main metabolites contributing to the separation were identified with higher values of correlation coefficients (denoted by a red colouration in (Figure 2C,D). Moreover, 17 and 16 variables of interest with VIP values > 1 were identified for high- and low-frequency conditions, respectively. Of note, only one variable of interest differs between the HF and LF condition: the 5.86 ppm urea bucket stands out as a discriminator only in the HF NMR dataset acquired, and this is not unexpected in view of its detectability only at the latter operating frequency.

From these variables, 5 (LF) and 6 (HF) potentially discriminant metabolites were identified. Indeed, glucose is increased in T2D patients, whilst levels of citrate, creatinine, indoxyl sulfate, urea (only observed in HF analysis) and hippurate are significantly reduced (Figure 2C,D). With the exception of urea, the metabolites responsible for the separation observed between these clusters were similar in both datasets acquired. Model validity was assessed via permutation testing (1000 permutations, HF Q^2^ value *p* < 0.001; LF Q^2^ value *p* < 0.001) and ROC analysis (Figure 2E,F). ROC curves generated via Monte Carlo cross-validation (MCCV) and based on the PLS-DA strategy (using 2 latent variables) demonstrated that the overall mean classification success rate was 92% and 90% for the HF and LF ^1^H NMR models, respectively.

The most effective PLS models were those incorporating 25 bucket regions, the AUROC value obtained being 0.96 for spectra acquired by HF, and 0.94 when using LF NMR analysis (95% confidence intervals 0.81–1.00). Therefore, based on the AUROC values and the overall Q^2^ and R^2^ scores obtained above, both models revealed a highly effective discriminatory ability. In view of complications arising from the very prominent glucose resonances present in approximately one-half of the T2D urinary ^1^H NMR profiles, and the application of the CSN (constant sum normalisation) approach, we elected to perform a corresponding statistical analysis on the full dataset after removal of all glucose resonance bucket regions. Indeed, following the removal of these resonance regions, the dataset was again constant sum-normalized and Pareto-scaled. Briefly, the results acquired confirmed significantly diminished urinary concentrations of hippurate (*p* < 0.005 to < 0.05 for three bucket regions) and indoxyl sulfate (*p* < 0.007 to < 0.05, again for three bucket regions). This univariate analysis was performed on all remaining (non-glucose) resonances using false discovery rate (FDR)-corrected two-samples *t*-tests.

We also directly compared the mean TSP-normalised intensities of ‘bulk resonance bucketed’ ^1^H NMR signals between the HF 400 and LF benchtop 60 MHz spectrometers employed for this study in a univariate manner via a paired sample t-test, and found that the only significant ‘between-spectrometer’ difference observed was that between the δ = 2.89 ppm trimethylamine N(CH_3_)_3_ singlet resonance, this value being greater for the 60 MHz spectrometer (FDR-corrected *p* value 0.002). However, this difference can be rationalized by its close location to the relatively intense joint creatinine/creatine >N-CH_3_ signal (δ = 3.03 ppm), which at an operating frequency of only 60 MHz, will be expected to provide a significant contribution towards the intensity of that of TMA. For this analysis, a series of 12 pre-selected resonances (of signal-to-noise (STN) values ≥ 10) were compared, and the dataset was also generalized logarithmically (glog)-transformed and Pareto-scaled prior to analysis.

### 2.3. Linear Correlations of Glucose Levels with the Urinary Metabolome of Diabetic Patients

**Figure 3:**
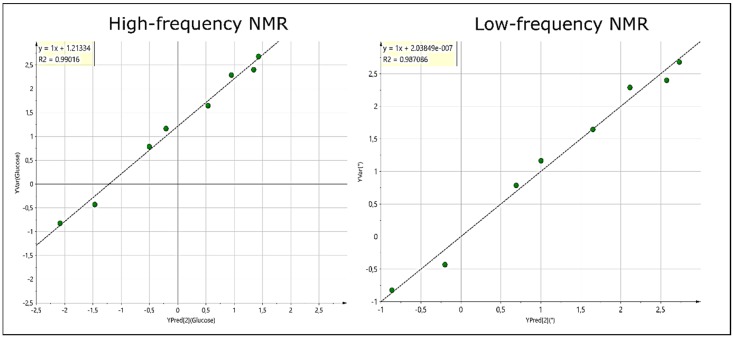
Transversal correlation studies between the T2D urinary metabolome and urinary glucose levels. PLS regressions were used to determine correlations between the glucose levels (Y actual values, on the *y*-axis) and the urinary metabolome of diabetic patients (depicted as Y predicted, on the *x*-axis) from spectra acquired by HF (left panel) or LF NMR analyses (right panel).

Potential relationships between the urinary levels of glucose, measured independently with a spectrophotometric GOD-PAP method as a clinical chemistry standard, and the urinary metabolomic profiles acquired were evaluated by conducting transversal correlation studies. These correlations were obtained by performing PLS regression (PLS-R) using only the non-glucose resonance NMR spectral regions of diabetic urinary samples included as the X-matrix, and the non-NMR-determined glucose levels as the Y-matrix; spectral regions of the glucose resonances (3.12–4.00, 4.15–4.35 and 5.10–5.40 ppm) were removed from the X-matrix prior to analysis, and the complete remaining dataset was then again constant sum-normalised and Pareto-scaled. It should also be noted that the glucose values vary greatly (spanning from 0.15 to 251 mmol/L), and hence were logarithmically-transformed to facilitate linear regression analysis. Two T2D samples with a glucose concentration value of 0 were excluded from the study. As shown in Figure 3, PLS regression lines for glucose levels correlate strongly with the urinary metabolome of all *n* = 8 T2D patients, with R² coefficients of 0.99 and 0.89 when using HF or LF spectra, respectively (Figure 3). On the basis of the VIP plots, variables with values ≥ 1 were selected as key ones related to urinary glucose variation. A total of 18 variables were determined as significant, and from these, 5 metabolites identified were found to be correlated with urinary glucose levels. Indeed, creatinine, alanine, citrate, lactate and N-acetylsugar/N-acetylamino acid resonances all appeared to be negatively correlated with urinary glucose levels. Hence, diabetic patients presenting with lower levels of urinary glucose display increased levels of hippurate, indoxyl sulfate, citrate and lactate levels, when compared to patients with high glucose values.

## 3. Discussion

Metabolomics is a powerful approach which, in the space of a few decades, has developed considerably, and HF NMR analysis is one of the most commonly applied techniques to perform metabolomics studies. However, despite adoption in several domains, the application of LF spectrometers for this purpose remains limited [8,11,13]. Here, we have reported metabolomics analysis comparisons between the ^1^H NMR profiles of T2D and control participant urine samples acquired by both high- and low-frequency NMR techniques. In our chemometrics analysis, T2D urine samples are readily distinguishable from those of healthy controls, and several metabolites are linked to this separation. Indeed, while glucose levels are higher in T2D patients, those of hippurate and indoxyl sulfate are lower in these participants. These results are observable in both HF and LF spectra, with OPLS-DA scores plots presenting highly similar spatial localizations of each sample at both operating frequencies, as well as corresponding discriminatory metabolites, with the exception of urea. Hence, under the same bucketing and normalisation conditions, it is possible to attain comparable results at both high and low operating frequencies. However, several limitations of this study should be considered, primarily, a probable insufficient sample size was involved, and the high levels of glucose present in some of the T2D urine specimens renders a facile multivariate metabolomics separation between the two conditions. The robustness of this approach still requires further testing on a large number of samples, and in a study investigating more non-glucose-based metabolic changes. Nevertheless, this challenges the paradigm of HF NMR applied in metabolomics, and also provides potential scope and insight for the development of new metabolomics strategies employing faster, cheaper and easier to use NMR techniques.

Poorly controlled diabetic patients regularly suffer from nocturia and polyuria, the former a condition in which patients awaken at least once during the night to void urine [14]. These lead to decreases in the concentrations of many urinary metabolites, together with wide ’between-participant’ variations in urinary excretion volumes. This may explain why T2D group downregulations in hippurate and indoxyl sulfate are also responsible for the ‘between-disease class’ distinction observed here, with significantly higher concentrations observed in the healthy control group. One approach to adjust for this would be to normalize the dataset using creatinine levels, which are usually excreted steadily in the urine. However, this was not possible in our study for the spectra acquired by LF NMR analysis, since the two buckets corresponding to this biomolecule’s resonances at δ = 3.06 and 4.06 ppm were subject to several overlap instances with other metabolites, i.e., those with resonance frequencies close to those of these signals. This is highlighted by the linear correlation observed between the integrals of the 1D versus 2D cross-peaks for creatinine, since the resulting correlation coefficient value is lower when compared to other corresponding plots obtained. This can be rationalised by considering several closely located metabolites signals, such as creatine and glucose, that are also likely integrated within the creatinine signal chemical shift region in 1D analysis, whilst integration of the 2D cross-peak signal serves to limit the superposition of resonances related to other metabolites. Furthermore, approximately 25% of diabetic patients suffer from kidney dysfunction [15], which is known to affect creatinine clearance [16], a phenomenon further hindering any creatinine normalisation strategies in metabolomics studies involving diabetic patients.

In the present study, urinary glucose levels were positively correlated with the non-glucose urinary metabolome of T2D samples. In particular, the abundance of several metabolites was downregulated based on glucose levels excreted in patient urine samples. High levels of glucose in the urine serve as commonly employed markers of poorly-controlled diabetes. These metabolites could correspond to the excretion of biomarkers of cellular or tissular dysfunctions, or defects linked to diabetes management. Further studies with larger sample sizes are therefore required to confirm the significant discriminatory metabolites identified in this case, since they may constitute valuable biomarkers for mechanistic studies, and the clinical monitoring of patients with type 2 diabetes. Furthermore, as noted above, multivariate correlation studies reveal that T2D patients presenting with lower levels of glucose display increased levels of hippurate, indoxyl sulfate, citrate and lactate, when compared to those with high glucose values. In our metabolomics analysis, OPLS loadings revealed that these urinary metabolites are at higher levels in the healthy control group, when compared with T2D participants. Therefore, this observation may suggest that glycaemically-controlled T2D patients with higher levels of these metabolites (and correspondingly lower urinary glucose levels) display a metabolomics profile more closely related to that of healthy control patients, and hence potentially reveals a better managed or controlled disease status.

The linear correlation analysis performed between the 1D and 2D intensities of glucose, bulk ring glucose and creatinine resonances showed strong correlations, except for that observed with creatinine, as noted above, which attests to the validity of 2D spectra acquired with the sequence “POWER COSY”. In addition, the acquisition of 2D ^1^H-^1^H NMR profiles clearly gives rise to an improvement in the resolution of the signals observed, and in particular allowed for the integration of the cross-peak signal of β-glucose, which was not possible in 1D spectra in view of overlap of its δ = 4.63 ppm anomeric proton signal with that of the residual water resonance in this spectral region. It should also be noted that apart from creatinine and glucose, the cross-peaks of other metabolites present at lower concentrations are not observable in the 2D spectra acquired. The sensitivity of the 2D spectra is lower than that of the 1D approach [17], and this is presumably why only the cross-peaks of the metabolites present in large quantities are observable. However, these spectra were acquired with only eight scans in only 30 min., and therefore increasing the number of scans may permit the observation of cross-peaks of several other metabolites. In addition, a metabolomics analysis of 2D COSY spectra acquired by HF has previously been developed [18,19], and these strategies could, in principle, also be applied to LF spectra, which could conceivably assist researchers in the context of overcoming the overlapping resonance issue encountered in 1D LF spectra, and also possibly increase the number of discriminatory metabolites discovered in such LF metabolomics investigations.

Problems encountered with the ^1^H NMR detection of urea at LF are explicable by the presence of unassigned doublet resonances detectable at this operating frequency. Hence, the removal of this signal may render urea analysis in such T2D urine samples possible. Since these signals are located close to the residual water signal, they may also be related to the solvent suppression sequence employed, and therefore further investigations are required to evaluate these effects. Unfortunately, such errors constitute a bias that must be considered when confirming the identities of ^1^H NMR signals and/or metabolomics investigations.

Finally, our results suggest that LF spectrometers could provide excellent, easy-to-use, compact and inexpensive tools to perform preliminary diagnostic analyses, either in laboratories or at external locations. Moreover, the quality of 2D spectra obtained within only a few minutes would broaden the horizon of its potential applications, such as drug analysis [20], or applications in the environmental [21] or nutrition fields [13]. As demonstrated here, 2D NMR spectral analysis could also contribute to personalised medicine and translational metabolomics in the areas of healthcare or dentistry. However, there remain a number of major drawbacks that limit their application in routine metabolomics analysis, such as low biomarker sensitivities and *J*-couplings which are spread over a much wider range of the total spectral width (for example, as much as 0.20 ppm for a triplet resonance with a *J* value of 6 Hz), phenomena which limit the number of detectable metabolites in a single sample. Therefore, these major issues must be addressed in the near future, in order to allow LF NMR diagnostic technologies to gain momentum in clinical applications.

## 4. Materials and Methods

### 4.1. Study Design

All samples in this study were collected with informed consent and approved by the appropriate Research Ethics Committee, specifically the Faculty of Health and Life Sciences Research Ethics Committee, De Montfort University, Leicester, UK (reference no. 1936). All participants were primarily provided with participant information sheets (PISs), and were then required to sign a project consent form in the presence of a researcher witness. The PIS clearly informed those recruited that since their participation was voluntary, they had the freedom to withdraw from the investigation at any stage of the process. All participants were also requested not to consume any alcoholic beverages, nor any dietary sources known to affect the human metabolome, for 24 hr. prior to urine sample collection. Essentially, all ethics considerations were in accordance with those of the Declaration of Helsinki of 1975 (revised in 1983).

### 4.2. Urine Collection

Participants fasted for a minimum period of 12 hr. prior to sample collection. Urine samples were collected from healthy control (*n* = 15) and T2D patients (*n* = 10), in sterile, plastic universal containers (King Scientific, Huddersfield, UK). These specimens were then transported to the laboratory on ice, and centrifuged immediately (3500 rpm at 4 °C for 15 min.). The supernatants were finally stored at −80 °C prior to ^1^H NMR analysis.

### 4.3. Spectrophotometric Determination of Glucose

Glucose was quantified using the well-established glucose oxidase-peroxide/4-aminophenazone/phenol (GOD-PAP) spectrophotometric methodology, which is outlined in Percival et al. [11] A glucose calibration curve, of concentrations ranging from 0.50 to 2.5 mg/mL, were prepared in 0.90% (*w*/*v*) NaCl (glucose was purchased from Sigma-Aldrich Chemical Co., Gillingham, UK). A 0.50 mL volume of the GOD-PAP reagent was incubated at 37 °C (Stuart Scientific Incubator SI 19, Stone, UK) for 10 min. in a polystyrene cuvette (1.0 mL path length, Fisher Scientific, Loughborough, UK). Then, 5 µL of each calibration standard was added to the cuvettes and incubated for a further 10 min. at 37 °C. Cuvette solutions were homogenized and equilibrated for a further 10 min. at 37 °C. Subsequently, these solutions were analysed using a spectrophotometer (Evolution 60S, Thermo Fisher Scientific, Leicester, UK), at a wavelength of 510 nm. The same procedure described here was followed for urine samples, using 5 µL of urine in place of glucose calibration standard solutions. The calibration curve had an R^2^ value of 0.990.

### 4.4. Low-Frequency (60 MHz) ^1^H-NMR Analysis

Optimised sample preparation techniques for urine samples have been previously described elsewhere [11]. Briefly, ^1^H NMR spectra were acquired using a 60 MHz Magritek benchtop facility (Spinsolve Ultra Proton), operating at a frequency of 61.67 MHz. For the urine samples, a 90:10% (*v*/*v*) ratio of H_2_O:D_2_O was used, the latter also containing 0.05% (*w*/*v*) sodium 3-trimethylsilyl-(2,2,3,3-^2^H_4_)-1-propionate (TSP). Samples were prepared at an ambient temperature in 5-mm diameter NMR tubes. Sample parameters for the 1D ^1^H NMR analysis were as follows: 64 scans, acquisition time of 6.4 s, repetition time 10 s, and a pulse angle of 90°, as previously described [11] pectra were acquired using a one-dimensional presaturation (1D PRESAT) sequence, to allow for efficient saturation of the water signal, and the water-suppression pulse was placed during the relaxation delay (1 s).

A gradient-enhanced magnitude COSY experiment (pulse sequence cosy 1H-1H Power Cosy, supplied by Magritek GmbH) was employed for the acquisition of 2D ^1^H-^1^H spectra. Spectra were collected with 8192 datapoints in F2, and 256 points in F1, over a sweep width of 14 and 46 ppm respectively; 8 scans were performed per F2 value, along with 4 dummy scans and a receiver gain of 101. The acquisition time was 0.16 and 1.63 s for F1 and F2, and the free induction decay (FID) resolution was 0.61 and 7.81, respectively. Resulting ^1^H-^1^H COSY spectra were processed in Topspin 4.0.6 and ACD labs 12.0 software modules using standard approaches, with sine-squared apodization in both dimensions, and zero filling in F1 to yield a transformed 2D dataset of 8192 by 256 datapoints.

### 4.5. High-Frequency (400 MHz) ^1^H-NMR Analysis

Identical preparation techniques were used for the HF ^1^H NMR analysis. Sample parameters for the 1D *noesygppr1d* HF 400 MHz analysis are detailed elsewhere [11]. Briefly, samples were acquired using 128 scans, with a size of 32,768 over a spectral width of 12.11 ppm, using an acquisition time of 3.38 s. and a receiver gain of 128 on a 400 MHz NMR facility (Bruker Avance I, Coventry, UK), operating at 399.93 MHz. Solvent suppression was employed in order to diminish the signal arising from water at δ = 4.80 ppm. Samples were placed in 5 mm NMR tubes and were analysed at random using an autosampler.

### 4.6. Multivariate Metabolomics Analysis

The same preprocessing and multivariate analysis strategies were applied to both LF- and HF-acquired FIDs. For statistical analysis, FIDs were converted and imported into Bruker using the “JCONV” command. They were processed with the Bruker Topspin 4.0.6 software with a standard parameter set. Phase and baseline corrections were performed manually over the entire range of the spectra, and the δ scale was calibrated to 0 ppm, using the internal standard TSP. Optimized ^1^H-NMR spectra were imported into ACD Labs 12.0 (Toronto, ON, Canada). Fixed bucket spectral intensities were then normalized to total intensities (also known as constant sum normalisation), and reduced to integrated regions of equivalent spectral width (0.04 ppm), corresponding to the 0.50–10.00 ppm range. The reduced and normalized NMR spectral data were imported into SIMCA (version 13.0.3, Umetrics AB, Umea, Sweden). Pareto-scaling was applied to bucketed data, and a multivariate metabolomics analysis, including principal component analysis (PCA) and an orthogonal partial-least squares discriminant analysis (OPLS-DA) were performed. SIMCA was used to generate all PCA, OPLS-DA and partial least-squares regression (PLS-R) model analyses, and corresponding plots. PCA was used to detect possible outliers and visualise intrinsic clusterings within the dataset, whilst OPLS-DA maximized the separation and facilitated the graphic visualization of differences and similarities between groups. The quality of OPLS-DA models was determined by goodness of fit (*R*^2^) values; the predictability of these were calculated on the basis of the fraction correctly predicted in a one-seventh cross-validation process (involving *Q*^2^ determinations). Ordinary linear regression approaches were performed using Excel 2016 (Microsoft Corporation, Redmond, WA, USA). All univariate statistical analyses were conducted using the freely available web-tool Metaboanalyst (*metaboanalyst.ca*).

By using the web-based analysis tool, Metaboanalyst (www.metaboanalyst.ca), receiver operating characteristic (ROC) curves were generated to assess the robustness of the models. ROC analyses were based on PLS-DA models, as classification methods with 2 latent variables (i.e., major orthogonal components). Model sensitivity and specificity were calculated from the ROC confusion matrix (generated on the basis of the average of predicted class probabilities of each sample across 100 cross-validations). ROC curves were generated by Monte Carlo cross validation (MCCV), using balanced sub-sampling. In each MCCV, two thirds (2/3) of the samples were used to evaluate feature importance. The top 2, 3, 5, 10 … 100 (max) important features were then used to build appropriate classification models, which were then validated on the one-third of the samples that were left out. This procedure was repeated multiple times to calculate performance and associated 95% confidence intervals (CIs) for each model system.

### 4.7. Metabolite and Discriminatory Biomarker Identification

From PLS-DA and OPLS-DA loading plots, metabolites with higher loadings values were identified. Signals with variable importance in projection (VIP) values ≥ 1 were considered as significant, and further validated using two-sample *t*-tests within Metaboanalyst. Metabolite identification was then performed using the open-access database NMRsuite 8.1 (Chenomx inc., Edmonton, AB, Canada), the free web-based tool Human Metabolome DataBase (HMDB) (http://www.hmdb.ca), and a full consideration of substantial chemical shift and coupling constant values available throughout the literature.

## 5. Conclusions

NMR spectroscopy is a powerful and reliable tool to assess the molecular compositions of biosamples using multivariate metabolomics strategies. In this study, we assessed the capabilities of a metabolomics analysis of data acquired on a LF benchtop NMR spectrometer using both 1D and 2D strategies, and compared results from these investigations to those obtained on a much higher frequency spectrometer. We also evaluated the strengths and limitations of LF 2D COSY spectra acquired for this purpose. Overall, our findings highlight the ability of a LF NMR approach to successfully reproduce metabolomics results achieved using a HF option. Moreover, the 2D COSY spectra of urinary diabetic samples gave rise to an improved level of identification and sensitivity, in relation to the resonances affected or partially overlapped by the more prominent glucose ones observed in 1D spectra. Further improvements in the numbers of metabolites detectable, and also the sensitivity of such LF NMR techniques, will, however, be achievable with greater numbers of acquisitional ^1^H-^1^H 2D COSY scans. Finally, development and applications of LF spectrometers are rapidly gaining momentum, and other metabolomics studies are very likely to be performed on this technique in the near future.

## Supplementary Materials

The following are available online at https://www.mdpi.com/2218-1989/10/4/155/s1, Figure S1: Urinary spectra of diabetic patient acquired at 60 (red, upper panel) and 400 MHz (blue, lower panel). Table: ^1^H NMR assignments for identified metabolites. Legend: s: singlet, d: doublet, t: triplet, m: multiple, dd: doublet of doublets.
