# Supplementary material for: Benchtop Low-Frequency 60 MHz NMR Analysis of Urine: A Comparative Metabolomics Investigation
Source: Metabolites. 2020 Apr 16;10(4):155. doi: 10.3390/metabo10040155 (PMC7240954; doi:10.3390/metabo10040155)
Supplement: Supplementary file 1 [file metabolites-10-00155-s001.pdf]

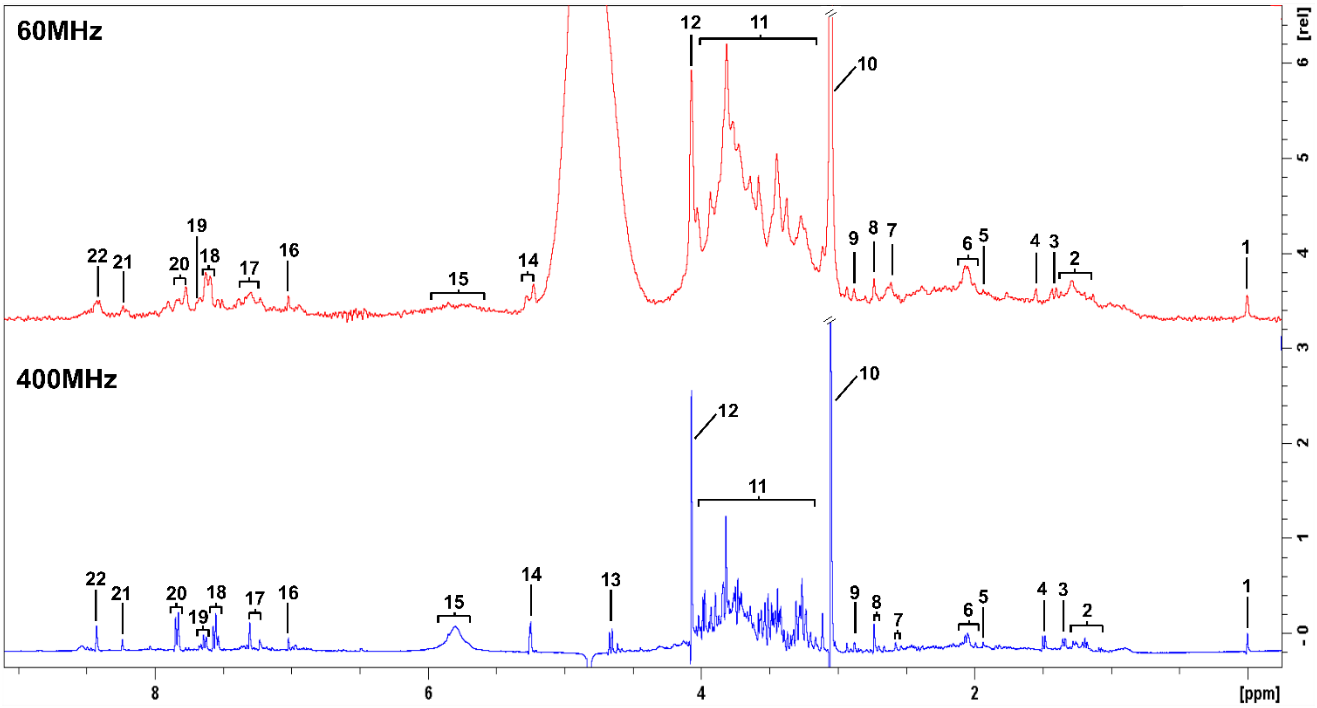

Number	Chemical Shift (δ/ppm)	Multiplicity	Assignment
1	0.00	s	TSP-C<u>H</u>3
2	1.00-1.30	Various:<br>t<br>d<br>d	Branched Chain Amino Acids :<br>Leucine<br>Isoleucine<br>Valine
3	1.34	d	Lactate
4	1.45	d	Alanine
5	1.95	d	Acetate
6	2.05	d	N-Acetyl
7	2.52	d	Citrate
8	2.70	d/s	Citrate/Dimethylamine
9	2.90	s/	Trimethylamine
10	3.05	s	Creatinine
11	3.20-3.90	various	Bulk Chain Glucose Resonances
12	4.06	s	Creatinine
13	4.62	d	β-glucose
14	5.25	d	α-glucose
15	5.80	broad	Urea
16	7.01	s	Histidine
17	7.20	m	Indoxyl sulfate
18	7.55	t	Hippurate
19	7.62	t	Hippurate
20	7.80	d	Hippurate
21	8.31	s	Histidine
22	8.45	s	Formate

Supplementary Figure 1: Urinary spectra of diabetic patient acquired at 60 (red, upper panel) and 400MHz (blue, lower panel). Table: ^{1}H assignments for identified metabolites. Legend: **s**: singlet, **d**: doublet, **t**: triplet, **m**: multiple, **dd**: doublet of doublets
